# Biological functions at high pressure: transcriptome response of *Shewanella oneidensis* MR-1 to hydrostatic pressure relevant to Titan and other icy ocean worlds

**DOI:** 10.3389/fmicb.2024.1293928

**Published:** 2024-02-13

**Authors:** Judy Malas, Daniel C. Russo, Olivier Bollengier, Michael J. Malaska, Rosaly M. C. Lopes, Fabien Kenig, D'Arcy R. Meyer-Dombard

**Affiliations:** ^1^Department of Earth and Environmental Sciences, University of Illinois Chicago, Chicago, IL, United States; ^2^Nantes Université, Univ Angers, Le Mans Université, CNRS, Laboratoire de Planétologie et Géosciences, LPG UMR 6112, Nantes, France; ^3^Jet Propulsion Laboratory, California Institute of Technology, Pasadena, CA, United States

**Keywords:** high hydrostatic pressure, astrobiology, ocean worlds, Titan, Europa, transcriptome, biosignatures, food safety

## Abstract

High hydrostatic pressure (HHP) is a key driver of life's evolution and diversification on Earth. Icy moons such as Titan, Europa, and Enceladus harbor potentially habitable high-pressure environments within their subsurface oceans. Titan, in particular, is modeled to have subsurface ocean pressures ≥ 150 MPa, which are above the highest pressures known to support life on Earth in natural ecosystems. Piezophiles are organisms that grow optimally at pressures higher than atmospheric (0.1 MPa) pressure and have specialized adaptations to the physical constraints of high-pressure environments – up to ~110 MPa at Challenger Deep, the highest pressure deep-sea habitat explored. While non-piezophilic microorganisms have been shown to survive short exposures at Titan relevant pressures, the mechanisms of their survival under such conditions remain largely unelucidated. To better understand these mechanisms, we have conducted a study of gene expression for *Shewanella oneidensis* MR-1 using a high-pressure experimental culturing system. MR-1 was subjected to short-term (15 min) and long-term (2 h) HHP of 158 MPa, a value consistent with pressures expected near the top of Titan's subsurface ocean. We show that MR-1 is metabolically active *in situ* at HHP and is capable of viable growth following 2 h exposure to 158 MPa, with minimal pressure training beforehand. We further find that MR-1 regulates 264 genes in response to short-term HHP, the majority of which are upregulated. Adaptations include upregulation of the genes *argA, argB, argC*, and *argF* involved in arginine biosynthesis and regulation of genes involved in membrane reconfiguration. MR-1 also utilizes stress response adaptations common to other environmental extremes such as genes encoding for the cold-shock protein CspG and antioxidant defense related genes. This study suggests Titan's ocean pressures may not limit life, as microorganisms could employ adaptations akin to those demonstrated by terrestrial organisms.

## 1 Introduction

Physical and chemical parameters such as acidity and alkalinity, salinity, temperature, and pressure are known to shape the evolution of life on Earth. These parameters place limitations on life, though the upper and lower limits of habitability have been expanding through the study of extreme environments and laboratory experiments (Schleper et al., 1995; Takai et al., 2008; Kusube et al., 2017). Indeed, life on Earth has been found to occupy a wide variety of “extreme” environmental niches (Harrison et al., 2013; Picard and Daniel, 2013; Ando et al., 2021; Shu and Huang, 2022). Many such environments are seen as analogs to a large selection of planetary contexts that could harbor yet-to-be-discovered extraterrestrial life (Merino et al., 2019).

We now have ample evidence for the existence of global, liquid water oceans beneath the ice shells of several planetary bodies in our solar system (Khurana et al., 1998; Hussmann et al., 2006; Waite et al., 2009; Iess et al., 2012). The icy ocean moons Titan, Europa, and Enceladus represent planetary systems that may have possessed all the fundamental properties required for life - water, energy, and organics - over extended periods of time (Vance et al., 2018; Hand et al., 2020). While the primary requirements for life may be met inside these moons based on current models of their interior, the subsurface oceans expected on larger icy worlds could still present several physical challenges to life, notably high hydrostatic pressure (HHP). For example, models of the hydrosphere of Europa estimate its ocean exists at pressures between 50 MPa, at the base of the overlying ice shell, and 250 MPa, at the top of the underlying rocky mantle (Vance et al., 2016, 2018; Weber et al., 2023). Part of that pressure range already exceeds the pressure at Challenger Deep in the Marianas Trench, the highest pressure deep-sea habitat explored on Earth (~11,000 m or 110 MPa) (Jannasch and Taylor, 1984; Stewart and Jamieson, 2019). The situation is even more extreme in the case of the larger moon Titan, whose subsurface ocean may experience pressures between 150 MPa (below an ice crust 80 to 100 km thick) and 800 MPa (Vance et al., 2018; Sotin et al., 2021), values 1.5 to 8 times above that of all previously explored life-supporting environments within Earth's ocean (Picard and Daniel, 2013).

Although life on Titan, Enceladus, or Europa would possess a different evolutionary history than life on Earth, life ultimately exists within the bounds of physical and chemical principles (Cockell, 2017). Therefore, understanding the effects of elevated hydrostatic pressure on biological systems is beneficial for understanding any planetary system where pressure is a fundamental environmental variable. As convergent evolution, or independent evolution of similar phenotypes under similar constraints, is ubiquitous on Earth (e.g., Agrawal, 2017), it could be expected that putative life on other planetary bodies would follow similar strategies. Therefore, the adaptive mechanisms adopted by life on Earth could provide precious hints at the biological processes involved at HHP in other worlds.

The primary physical challenge that HHP enacts on living organisms is a decrease in volume of the system, as governed by Le Châtelier's principle (Le Châtelier, 1884). HHP causes protein denaturation, alteration of protein functions, loss of membrane fluidity, loss of motility, and suppressed transcription/translation (reviews in Oger and Jebbar, 2010; Meersman et al., 2013; Mota et al., 2013; Silva et al., 2014). Despite these challenges, life has evolved to withstand high pressures, and organisms known as piezophiles have specific adaptations that allow them to grow more efficiently under HHP than at atmospheric pressure (e.g., Yayanos et al., 1979; Yayanos, 1995; Masanari et al., 2014; Peoples et al., 2020; Zhang et al., 2021). Adaptations to HHP identified from previous studies of piezophiles include increased protein flexibility (Martinez et al., 2016), increased abundance of heavy metal resistant genes (Zhang et al., 2021), and specialized chemotaxis, motility, and respiration systems (Michoud and Jebbar, 2016; Peoples et al., 2020). The currently demonstrated growth limit at HHP is 140 MPa (Kusube et al., 2017), just below the 150 MPa pressure expected at the top of Titan's ocean.

The ability to grow more efficiently at high pressures is considered a highly specialized evolutionary trait (Yayanos, 1986; Bartlett, 1992; Zhang et al., 2021). Nonetheless, several reports have indicated that non-piezophiles, such as *Shewanella oneidensis* and *Escherichia coli*, can survive much higher pressures, up to 1.5 GPa for short periods of time (15 min) (Hauben et al., 1997; Griffin et al., 2011; Vanlint et al., 2011; Hazael et al., 2014). The molecular basis for the survival of these non-piezophiles, particularly *S. oneidensis*, at Titan-like pressures is largely unknown (Macdonald, 2021a). Studies of adaptation mechanisms for other non-piezophilic bacteria at pressures above 150 MPa are few (Bowman et al., 2008; Duru et al., 2021; Zhu et al., 2021) and some focus only on the screening of a few genes of interest (Aertsen et al., 2004, 2005). Most studies exploring pressures from 100 MPa to 1 GPa focus on experimental factors influencing microbial inactivation of pathogens for safe food and beverage consumption (*reviewed in* Podolak et al., 2020). To elucidate the mechanisms of survival at Titan-relevant pressure, we conducted a study of the HHP induced transcriptome response of *Shewanella oneidensis* MR-1 at 158 MPa. Consistent responses of non-piezophiles to Titan-relevant HHP, mirroring those of piezophiles, may suggest a universal strategy among terrestrial microorganisms to extreme pressures.

## 2 Materials and methods

### 2.1 Bacterial growth conditions

**Figure 1:**
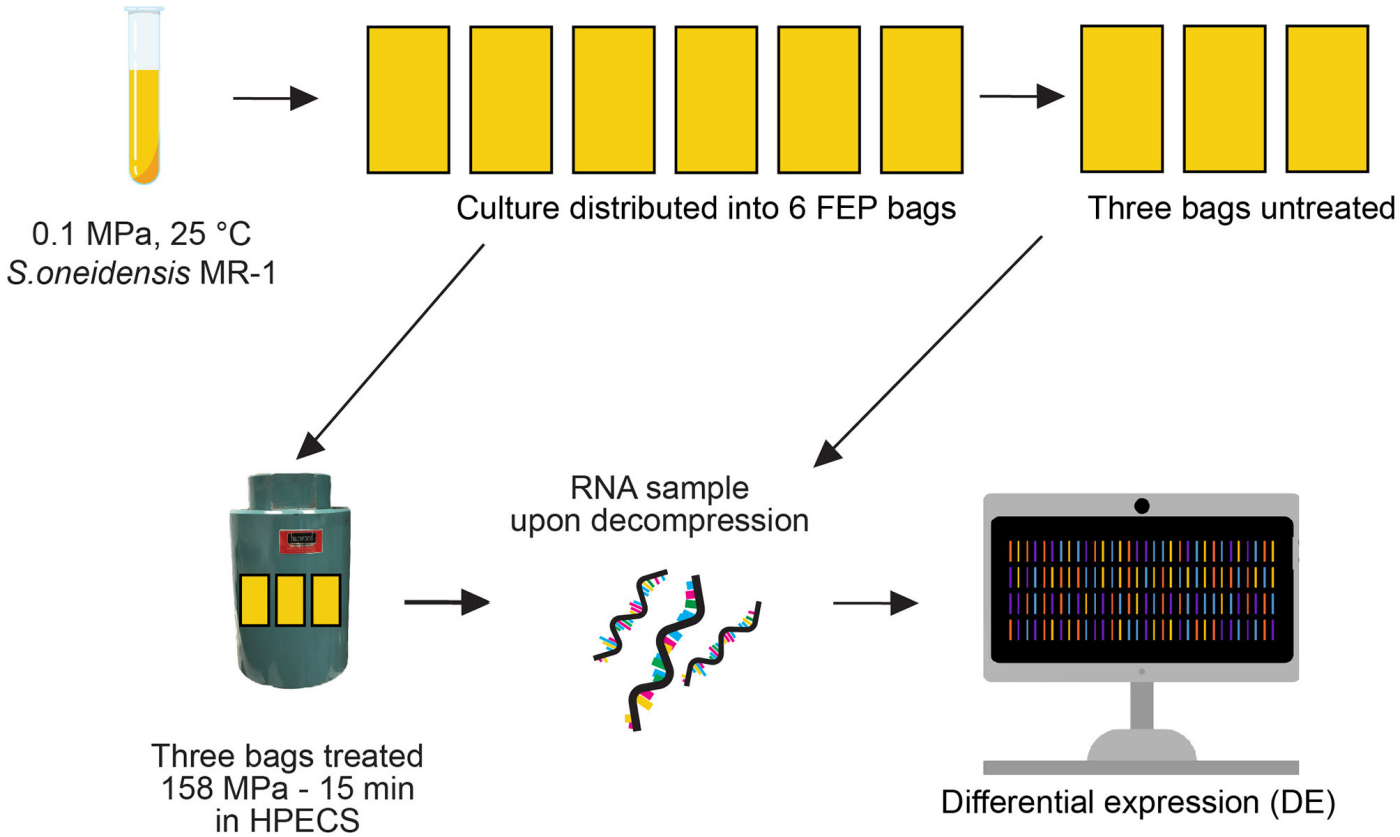
Experimental setup. The 158 MPa 15 min experiment was conducted in triplicate in the High Pressure Experimental Culturing System (HPECS). The teal cylinder is the Cell Body Vessel wherein samples are subjected to elevated hydrostatic pressure.

*Shewanella oneidensis* MR-1 was purchased from the American Type Culture Collection (ATCC), Product No. 700550. The freeze dried culture was reconstituted and grown anaerobically at 0.1 MPa (atmospheric pressure) in tryptic soy broth (per 500 mL deionized water: 8.5 g tryptone, 1.5 g soytone, 1.25 g dextrose, 2.5 NaCl, 1.25 g K_2_PO_4_; final pH 7.3). Prior to pressure treatment, cultures were grown to early stationary phase (~1 x 10^8^ cell per mL; Figure 1) in glass culture tubes (N_2_ headspace). Fluorinated Ethylene Propylene (FEP) bags were custom made with an impulse heat sealer and were sterilized prior to use by autoclaving for 55 min at 121°C. *S. oneidensis* culture was transferred into the 1.5 by 3.5 inch FEP bags using a sterile syringe and needle within an anaerobic chamber (95% N_2_, 5% H_2_) to maintain anoxic culture conditions. Following the transfer, the bags were flattened to expel any excess gas and then clamped to provide a temporary seal. After removal from the anaerobic chamber, each clamped bag received its final heat seal. Stress and pressurization tests were carried on several test bags to ensure the heat seals were able to withstand the pressure cycles of the experiments. While the FEP bags proved effective in that regard, their relative stiffness complicated the filling process, resulting in variable load volumes of 2 to 5 mL per bag.

### 2.2 Pressure treatment

Two pressure conditions were tested: 158 MPa for 15 min (Figure 1), and 158 MPa for 2 h (Supplementary Figure S1). Each of the two pressure conditions was tested in triplicate (giving a total of six experiments). For the 15 min experiments, a previously untreated *S. oneidensis* culture was subjected to pressure treatment. Cells in early stationary phase were utilized as bacterial cells in this phase have previously been found to be more HHP resistant than cells in exponential phase (e.g., Griffin et al., 2011). For each experiment, six FEP bags were filled with these prepared *S. oneidensis* cultures. To ensure that the concentration of cells (per mL of loaded culture) was identical in all bags of any given experiment, a single culture per experiment was used to load all six bags. Three of the six FEP bags filled with *S. oneidensis* cultures were subjected to the pressure treatments, while the other three were kept at ambient pressure (0.1 MPa) to serve as untreated controls.

A high pressure “survivor” strain was used as inoculum for the 2 h experiments (Supplementary Figure S1). This survivor culture was obtained through exposure to three successive pressure cycles, each at 158 MPa but of increasing duration (15 min, 1 h, 2 h), and each followed by recovery at ambient pressure for 48 h without re-inoculation or dilution. After recovery, the pressure treated cultures were re-inoculated into fresh TSB media (1:1000 dilution) and grown to early stationary phase (20−25 h).

Hydrostatic pressure was applied using a High Pressure Experimental Culturing System (HPECS) (Meyer-Dombard et al., 2024), manufactured by Harwood Engineering Company, Inc. (Walpole, MA). In this setup, a Harwood Engineering Company, Inc. Manganin^®^ Cell Body Vessel containing the experimental bags is connected to an Air Operated Pressure Panel and filled with a hydraulic fluid (a half-and-half mixture of water and ethylene glycol) prior to closure and pressurization. Pressurization of the hydraulic fluid in the system is achieved using a set of air pumps driving a Harwood A-2.5-J intensifier. Bags were pressurized at an average rate of 46.8 ± 16.4 MPa/min. The pressure stabilized at 158.2 ± 0.7 MPa across all six experiments within a minute. Target pressure was held for 15 min or 2 h, with pressure fluctuations remaining below 1 MPa. At the end of each experiment, the system was depressurized manually at an average rate of 45.1 ± 17.7 MPa/min. As the pressurization and depressurization steps involve some manual operations, a more rigorous regulation of these rates was not possible.

### 2.3 RNA extraction and sequencing

Upon decompression, the outside of the FEP bags were washed in tap water and soap (to get rid of the hydraulic fluid) before being sterilized with isopropanol/water (70% v/v). The sterilized bags then were punctured with a needle attached to a syringe. The contents of two FEP bags, about 2 mL−4 mL of culture, were transferred to a glass tube filled with 6 mL of RNAprotect Bacteria Reagent (QIAGEN, catalog #76506) with a headspace of nitrogen gas. The RNAprotect reagent stabilizes the RNA immediately and the nitrogen headspace ensures that the sample remains anoxic during sampling. The process of preservation in the RNAprotect reagent took 12 min−15 min per experiment after decompression. The RNAprotect reagent and culture mixture was then transferred into a Falcon tube and centrifuged at 2200 xg and 4°C for 20 min to obtain cell pellets. Simultaneously, two FEP bags that were not subjected to pressure were sampled identically. One HHP treated and one control FEP bag were kept sealed for observation and incubated at 25°C and 0.1 MPa. Pelleted samples were then frozen at −40°C until RNA extraction.

RNA was extracted with an RNeasy^®^ PowerBiofilm^®^ Kit (QIAGEN, catalog #25000–50). Sample cleanup and sequencing was conducted at Rush University Medical Center Microbiome Core Facility. The samples were purified using RNeasy MinElute Cleanup Kit (QIAGEN, catalog # 74204) along with RNase-Free DNase Set (QIAGEN, catalog # 79254) prior to library preparation. RNA quality was assessed with an Agilent TapeStation 4150 device. The RNA integrity number equivalent (RIN^e^) is a value between 1 and 10, from lowest to highest quality, that represents the degree to which the RNA has been degraded (Schroeder et al., 2006). The RNAseq libraries were prepared using the NEXTFLEX^®^ Rapid Directional RNA-Seq Kit 2.0 (catalog #NOVA-5198–03). Short read 2x150 bp sequencing was conducted on an Illumina NovaSeq6000 instrument. Raw data files were deposited to the Sequence Read Archive (SRA) and are available under BioProject accession number PRJNA1010834.

### 2.4 DNA extraction and genome sequencing

DNA was extracted from *S. oneidensis* MR-1 grown at 0.1 MPa and 25°C to early stationary phase. The culture was centrifuged at 2200 xg for 20 min to obtain pellets. Pellets were stored at −40°C until extraction. DNA extraction was performed with a DNeasy^®^ PowerLyzer^®^ PowerSoil^®^ Kit (QIAGEN, catalog # 12855–50), according to the manufacturer's instructions. Sequencing was conducted at Rush University Medical Center Microbiome Core Facility. Short read 2x150 bp sequencing was conducted on an Illumina NovaSeq6000 instrument. Raw data files were deposited to the SRA and the assembled and annotated genome was deposited to GenBank under the accession JAYGGX000000000. All data files are available under BioProject accession number PRJNA1010834.

### 2.5 Assessment of survival and viability

Aliquots from pressure treated and untreated FEP bags were preserved in 4% paraformaldehyde (PFM) for cell counting. Cell counts of DAPI (4′,6-diamidino-2-phenylindole, ThermoFischer, catalog #62248) stained cells were performed using a Leica DM5500 B fluorescence light contrast microscope. Cells were placed onto polycarbonate filters and stained with DAPI at room temperature for a minimum of 10 min. After staining, at least 10 fields of view were counted from each filter. Counts post-treatment were compared to cell counts pre-treatment and to untreated control from the same starting culture. Sealed bags were assessed visually for turbidity increase, which ensured that post-experiment viability was not a result of laboratory post-sampling contamination.

### 2.6 Data analyses

Raw DNA and RNA sequence reads were quality checked with FastQC (v 0.11.9, Andrews, 2010) and trimmed with Trimmotatic (v 0.39, Bolger et al., 2014). Genomic DNA was assembled with SPADES (v 3.15.3, Bankevich et al., 2012). Assembly quality was assessed with QUAST-LG (v 5.2.0, Mikheenko et al., 2018). The assembled genome was annotated with ProKKa (v 1.14.5, Seemann, 2014). Clusters of Orthologous Genes (COGs) identifiers were assigned via ProKKa. BlastKOALA (v 3.0) was used to assign K numbers to the genome corresponding to the Kyoto Encyclopedia of Genes and Genomes (KEGG) pathways (Kanehisa et al., 2016). Short-read RNA sequences were aligned to the protein coding sequences (CDS) of the assembled genome with STAR (v 2.7.9a, Dobin et al., 2013) and transcript counting was conducted with StringTie (v2.1.7, Pertea et al., 2015). The prepDE.py command was used within StringTie to produce a matrix of counts per gene which was used for statistical and differential expression analyses in the package EdgeR (3.36.0, Robinson et al., 2010). Transcripts were normalized using the trimmed mean of M values normalization (TMM) in EdgeR to account for compositional biases. Genes with a sum below 100 counts per million across two samples were filtered and removed from further analyses. Counts were subsequently log transformed, and hereafter log-2-counts per million are referred to as CPM. The “exactTest” function in EdgeR was used to calculate differential expression between groups. Adjusted *p-*values were calculated by Benjamini–Hochberg false discovery correction (FDR) of 5 % for all methods. Genes with adjusted *p* < 0.05 were and log-2-fold change (LFC) >1 or < −1 were considered differentially expressed.

## 3 Results

### 3.1 Viability post HHP treatment

**Figure 2:**
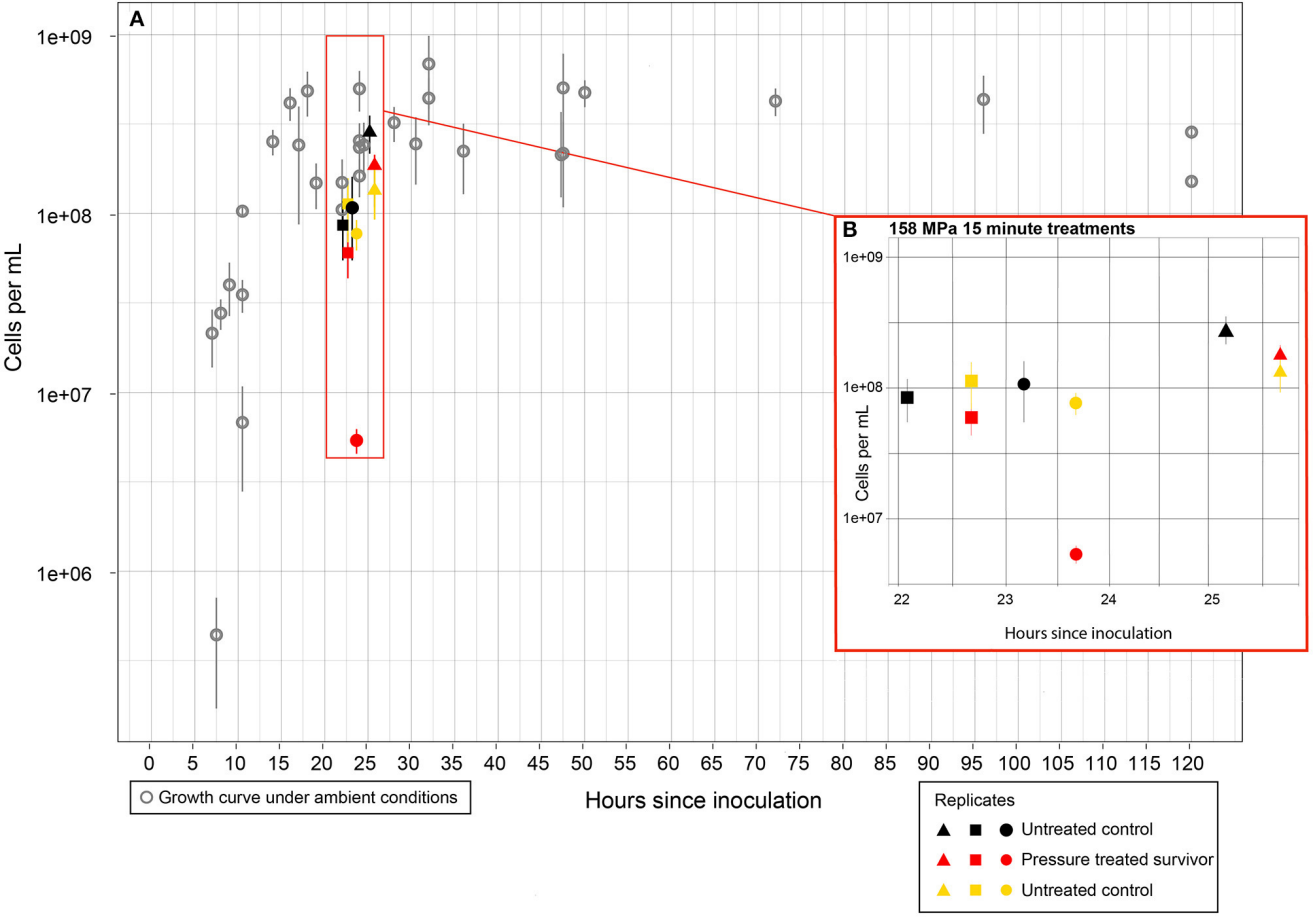
Cell counts of DAPI stained cells for the 150 MPa−15 min experiments. Error bars represent standard deviation about the mean least 10 fields of view were counted from each filter. **(A)** Gray open circles represent growth curve for *S. oneidensis* grown anaerobically in glass culture tubes under ambient conditions (0.1 MPa, 25°C) in tryptic soy broth. Black, yellow, and red shapes represent cell counts of cultures sampled from FEP bags. **(B)** Cell counts for experimental cultures. Cultures were transferred into FEP bags and cell counts were conducted at the start of the experiments (black shapes). FEP bags were either maintained at ambient conditions (yellow shapes) or subjected to 158 MPa treatment for 15 min (red shapes). Closed circles, squares, and triangles are experimental replicates of the treatment. Time between counts within each experiment includes 15 min treatment plus time for decompression until preservation of the samples in PFM.

Cell viability was assessed in two ways: (1) intact cell counts taken before and after high-pressure treatment and compared to untreated control (Figure 2; Supplementary Figure S2) and (2) qualitative visual estimation of turbidity changes within sealed, unopened HHP treated bags (Supplementary Figure S3).

#### 3.1.1 Condition 1: 158 MPa for 15 min

All the samples that were subjected to the 15 min HHP treatment were viable after recovery. In those samples, intact cells were observed (Figure 2), and turbidity of the cultures in the sealed bags increased within 24 h post-treatment (data not shown). Two of the three replicates from the 15 min experiments maintained cell numbers comparable to that of the untreated control; cell count for the third treated replicate decreased by an order of magnitude but still maintained >10^6^ intact cells/mL (Figure 2). RNA was recovered from all samples (HHP treated and unpressurized controls for comparison) (Supplementary Figure S4A). The RIN^e^ as determined from the TapeStation analysis was at least 6.9 for all 15 min experiment replicates, for both the untreated controls and HHP treated samples (Supplementary Figure S5).

#### 3.1.2 Condition 2: 158 MPa for 2 h

After exposure of the cell cultures to 158 MPa for 2 h, the number of intact cells in two of the three replicates remained similar to that of the untreated control (Supplementary Figure S2). Turbidity increased within 48 h of decompression for these two replicates. The HHP treated cultures of the third replicate appeared non-viable, as no intact cells were counted from the two bags that were opened, and turbidity in the remaining sealed bag did not increase in the following days. Although cell numbers for the two viable replicates were not significantly reduced as compared to the untreated controls, RNA concentrations recovered from the HHP treated samples were too low for sequencing (Supplementary Figure S4B). The RNA concentration from the 2 h untreated control bags also were lower than the RNA concentrations of the 15-min untreated controls. For both treatment conditions, cells for experimentation were grown to early stationary phase. Due to the longer experimental treatment, the untreated cultures of the 2 h treatment may have passed “early” stationary phase and started to become dormant, thus limiting the RNA yield.

After the turbidity had increase within the remaining sealed bags from the viable replicates, RNA sampling was performed again 48 h after decompression. This second phase of RNA sampling resulted in concentrations two orders of magnitude higher (Supplementary Figure S4C), reaching levels comparable to the RNA concentrations from the untreated control. However, after 48 h, the RNA^e^ of the untreated control samples from these two experiments was of lower quality (RIN^e^ of 3.3 and 1.4; Supplementary Figure S5), indicating that RNA had degraded and that the cell culture was likely in the “death” phase. Therefore, the 2 h experiments were not included in the differential expression analyses as there was no appropriate baseline for comparison.

### 3.2 Effect of 15 min HHP treatment on gene expression of *S. oneidensis*

RNA sampled immediately after decompression from the 158 MPa, 15 min treatment was mapped to the assembled genome. Short read sequences were assembled into 282 contigs, totaling 4,901,829 bp. The number of predicted CDS was 4,354 (Supplementary Table S1). For all six samples (3 treated, 3 control), between 79% and 82% of the RNA sequence reads were uniquely mapped to the genome. After quality filtering, a total of 1,204 genes were considered for differential expression between the pressure treated samples and their corresponding untreated controls. Principle components analysis (PCA) of total gene expression was conducted and showed the samples clearly separated by HHP treatment along PC1, which accounted for ~83% of the variance between samples, indicating that HHP treatment was the main influence on gene expression (Supplementary Figure S6). PC2 separated samples by replicate, but only explained 9% of the variance between samples. Common dispersion was calculated, and the biological coefficient of variation (BCV) was 0.21. Functional annotations were assigned using the Clusters of Orthologous Genes (COG) database and the Kyoto Encyclopedia of Genes and Genomes (KEGG) database. The full list of genes and results of the differential expression analysis are available (Supplementary Table S2).

**Figure 3:**
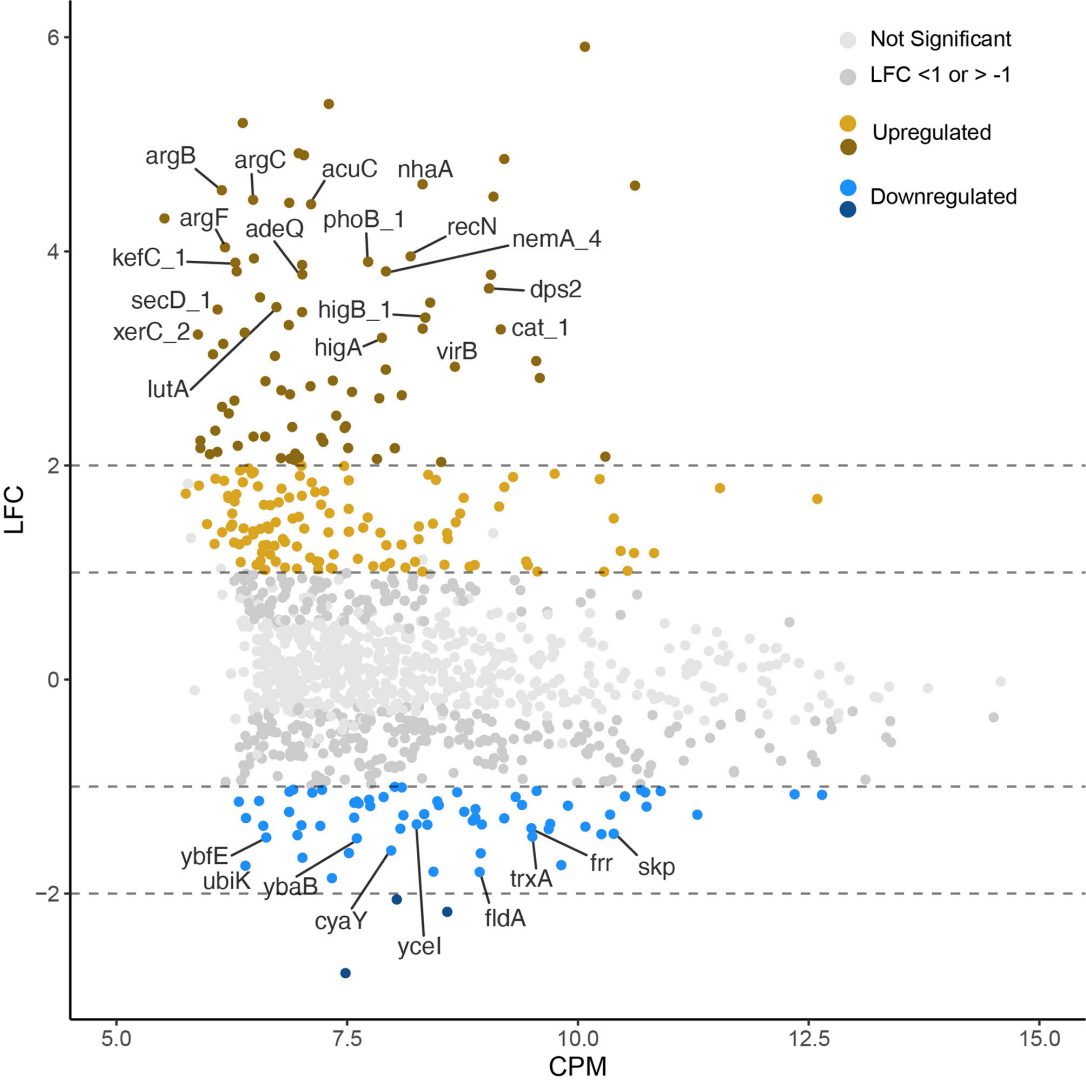
Log-2-fold-change (LFC) vs. log-2-counts per million (CPM) of each transcript (after exposure to 158 MPa for 15 min). Lighter gray points are not significantly up or downregulated (*p* < 0.05). Significantly up (*n* = 195) and downregulated (*n* = 69) genes above the LFC threshold are shown in yellow and blue, respectively. Dashed lines show absolute values of LFC >1 and >2 to highlight highly upregulated and downregulated genes. Gene labels were applied only to annotated transcripts with LFC > 3 or < −1.3.

**Figure 4:**
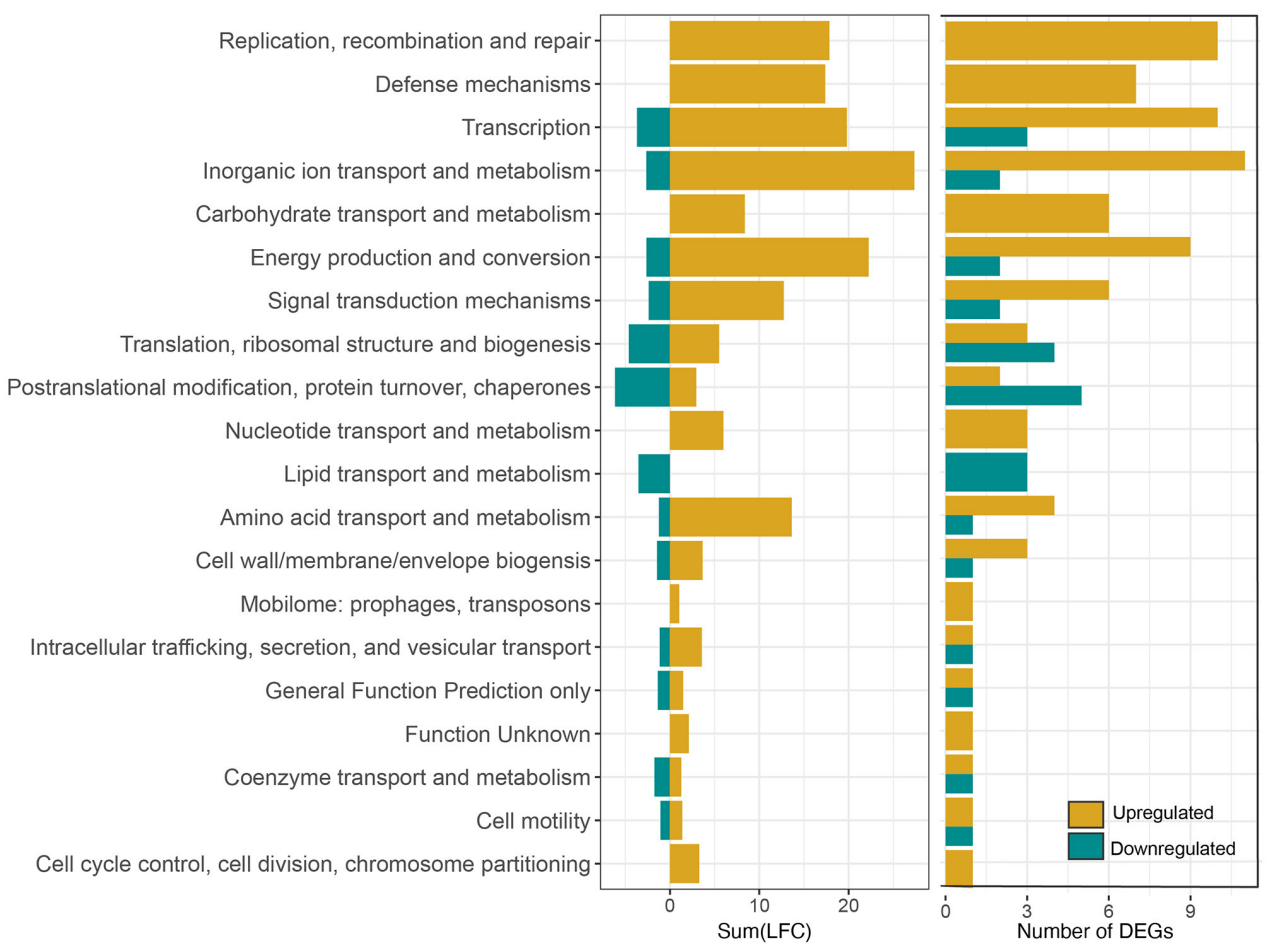
Summed log-2-fold-change (LFC) of all the DEGs in each COG category after exposure to 158 MPa for 15 min **(left)** and corresponding number of DEGs in each category **(right)**.

Out of the 1,204 genes examined, 264, constituting 22% of the tested genes, exhibited differential expression with a log-fold change (LFC) >1 or < −1 in the pressure-treated samples as opposed to the untreated controls. Among these 264 differentially expressed genes (DEGs), 195 were identified as upregulated, while 69 were downregulated. In addition, there were more highly upregulated genes (78) with LFC > 2 than highly downregulated genes (3) LFC < −2 (Figure 3). It is noteworthy that only 122 out of the total 264 DEGs were annotated, accounting for 46% of the identified DEGs. Functional categories assigned to the upregulated genes with the highest fold changes belong to the COG categories replication, recombination and repair (L), defense mechanisms (V), transcription (K), inorganic ion transport and metabolism (P), and energy production and conversion (C). Though there were significantly fewer downregulated DEGs, post translational modification, protein turnover, chaperones (O), translation, ribosomal structure, and biogenesis (J), and lipid transport and metabolism (I) are among the most downregulated COG categories (Figure 4).

#### 3.2.1 Ribosomes

The KEGG database identified fifty-three “large ribosome” related genes in the genome (i.e., 30S and 50S ribosomal proteins; Supplementary Table S3). About a third of these genes had lower expression in the pressure treated than untreated controls, including: *rpsP, rpsT, rplT, rplQ, rplX, rplN, rplY, rpmE, rpmG, rpmB, rpmF, rpmI*, and *rpsU*. The fold-change values for these ribosomal proteins were relatively low (LFC ranged from −0.3 to −1), however CPM values were relatively high (CPM > 10 for most genes). Only three genes were considered differentially expressed within the LFC threshold of < −1 (*rpmF, rpmB, rpmG)*. The ribosome recycling factor *frr* (LFC −1.39) was among the most downregulated genes (Figure 3).

#### 3.2.2 Energy production and conversion

Eleven genes were differentially regulated in this category (Figure 4). Genes encoding lactate utilization proteins A and B, *lutA* and *lutB*, were highly upregulated by 3.5 LFC and 2.4 LFC, respectively. Genes encoding NADPH and NADH reductase proteins, *azr* (LFC 2.1) and *azoR* (LFC 2.4), were also upregulated.

#### 3.2.3 Amino acid transport and metabolism

Upregulated genes in this category were related to arginine biosynthesis, including *argA, argB, argC*, and *argF*. These genes were among the highest upregulated genes, with LFC values of 2.7, 4.6, 4.5, and 4.0, respectively. The KEGG database identified 9 genes belonging to the arginine biosynthesis pathway in the genome, and in addition to the upregulated genes mentioned, *alaA* (Glutamate-pyruvate aminotransferase) and *glsA2* (Glutaminase 2) were detected in the transcriptome but not significantly differentially regulated.

#### 3.2.4 Transcription

The transcriptional regulators *lexA, cusR*, and *iscR* were upregulated (LFC 2.9, 2.2, and 1, respectively). The transcription termination factor gene *rho* was upregulated by LFC of 1.9 and had a high relative count (CPM of 9.7). The gene *cspG* (cold shock protein CspG) was upregulated by 1.7 LFC, while *cspC* (cold shock protein CspC) was downregulated by −1.3 LFC. Genes *ybaB* encoding for the nucleoid-associated binding protein and *dksA* encoding for RNA polymerase-binding transcription factor were downregulated by −1.4 LFC and −1.1 LFC, respectively.

#### 3.2.5 Replication, recombination, and repair

Several genes were highly upregulated (LFC ≥ 2) and highly expressed (CPM ≥ 7.5) including the genes *recN* (DNA repair protein), *topB* (DNA topoisomerase 3), *dinB* (DNA polymerase IV), and *dinG_1* (ATP-dependent DNA helicase). Significantly downregulated genes were not detected in this category (Figure 4).

#### 3.2.6 Cell membranes, lipids, and fatty acids

Genes encoding for fatty acid biosynthesis were detected in the pressure treated samples. The genes *fadL* (LFC −1.04) encoding for long-chain fatty acid transport protein, *acpP_3* (LFC −1.2) encoding for acyl carrier protein, and *dgkA* (LFC −1.3) encoding diacylglycerol kinase were moderately downregulated. Several lipid biosynthase-associated genes were detected in the transcriptome (FDR adjusted *p* < 0.05) but had absolute LFC values below the threshold of 1: *acpS* [Holo-(acyl-carrier-protein) synthase, LFC 0.8], *lpxM2* (Lipid A biosynthesis myristoyltransferase 2, LFC 0.8), *fabH2* [3-oxoacyl-(acyl-carrier-protein) synthase 3 protein 2, LFC 0.7]*, fabF_2* [3-oxoacyl-(acyl-carrier-protein) synthase 2, LFC 0.7], and *fabG_3* [3-oxoacyl-(acyl-carrier-protein) reductase, LFC −0.3]. Other expressed genes involved in fatty acid biosynthesis that were not significantly up- nor downregulated were: *fabH1, acpP_4, accA, fabB_2, fabG2, fabA, fabZ, plsB*, and *fadR* (Supplementary Table S2).

#### 3.2.7 Inorganic ion transport and metabolism

The COG category of “energy production and conversion” appears as the second most upregulated according to the sum of LFC values (Figure 4). Genes related to the K^+^, Na^+^, H^+^, Ca^2+^ cations transport and uptake, *nhaA* (LFC 4.6), *kefC_1* (LFC 3.9)*, nhaP2_2* (LFC 2.1)*, ktrB_2* (LFC 1.9), and *ktrA* (LFC 1.6) are highly upregulated, indicating a strong ionic stress on the cells. The genes *cusA_2* (LFC 2.7)*, cusA_1* (LFC 1.7) and *yfiH* (LFC 1.3), related to heavy metal stress and transport (primarily copper), were also upregulated.

#### 3.2.8 Defense mechanisms

Defense mechanism functional genes were highly upregulated (Figure 4). The DNA-binding ferritin-like protein involved in oxidative damage protection *dsp2* was upregulated by 3.7 LFC, the highest upregulated defense mechanism. Both the toxin *higB_1* (LFC 3.4)*/higB-2* (LFC 1.6) and antitoxin *higA* (LFC 3.2) components of the HigAB toxin-antitoxin module were upregulated.

## 4 Discussion

Understanding how non-piezophiles cope with high hydrostatic pressure (HHP) relevant to Titan could reveal shared responses among diverse life forms to pressure constraints. These shared molecular responses to pressure (i.e., molecular biosignatures) could be targeted in future planetary exploration missions to Titan and other subsurface ocean worlds. Oger and Jebbar (2010) summarized several mechanisms of HHP adaptation including: (1) fine tuning of overall gene expression; (2) adaptation of the structure of biomolecules to sustain HHP; and (3) expression of HHP-specific genes. While true piezophiles may possess specialized genes and biomolecules, the fine tuning of overall gene expression is employed by piezophiles and non-piezophiles alike in response to HHP (e.g., Amrani et al., 2014; Vannier et al., 2015; Michoud and Jebbar, 2016). However, most gene expression studies to date do not utilize Titan-relevant pressures. Responses of non-piezophiles to HHP relevant to Titan that are the same as those of piezophiles could indicate a ‘universal' response of terrestrial microorganisms to HHP.

We conducted short term HHP incubations to elucidate the mechanisms of survival at Titan-relevant pressures (*P* ≥ 150 MPa) in the model organism *S. oneidensis* MR-1. Two experimental conditions were tested: 158 MPa, 15 min exposure and 158 MPa, 2 h exposure. The data shown here suggest that *S. oneidensis* can mount a response to short-term pressure stress by “fine tuning” its gene expression changes after just 15 min at 158 MPa. Exposure of *S. oneidensis* to 158 MPa for 15 min brought about 264 differentially expressed genes (DEGs), the majority of which were upregulated (Figure 3). DEGs were involved in inorganic ion transport and metabolism, transcription, ribosomal structure, transcription, replication and repair, energy production and conversion, amino acid metabolism, defense mechanisms, among other biological functions (Figure 4).

### 4.1 Membrane and fatty acid adaptations

Lipid membranes are among the most pressure-sensitive biomolecules, and upon compression, lipids adapt to volume restriction by changing their conformation and packing, thus losing fluidity (Oger and Jebbar, 2010). This loss of fluidity leads to reduced transmembrane transport and loss of flagellar motility (Meganathan and Marquis, 1973). The regulation of motility related genes was observed in our studies (Figure 4). The *hag* (LFC −1.1) gene encoding the flagellin of *S. oneidensis* was downregulated. However, the gene *pomA_2* (LFC 1.4), encoding chemotaxis protein PomA was upregulated, indicating that *S. oneidensis* may have continued attempts to maintain its motility.

Genes involved in cell membrane biogenesis exhibited both upregulation and downregulation in response to HHP (Figure 4), suggesting *S. oneidensis* engaged in cell membrane reconfiguration during pressure treatment. In total, 11 of 20 genes responsible for type II fatty acid biosynthesis (White et al., 2005), were detected in the pressure treated samples (Supplementary Table S2). The gene *acpP_3*, which is essential for type II fatty acid biosynthesis (LFC −1.2) was downregulated but its homolog *acpP_4* was not significantly up- or downregulated (Supplementary Table S2), indicating fatty acid biosynthesis was not impaired during the HHP treatment.

To counter the loss of membrane fluidity, microorganisms respond by increasing the proportion of unsaturated, short chain, and branched chain fatty acids in their membranes (Tamby et al., 2022). Here, the *fadL* (LFC −1.04) gene, encoding a long-chain fatty acid transport protein was downregulated, while three other genes involved in fatty acids synthesis including *fabF_2, acpS* and *fabH2*, increased in expression (*p* < 0.05) but did not surpass the LFC threshold of 1 (LFC 0.75, 0.74, 0.83, respectively). In *S. oneidensis*, the condensing reaction for fatty acid synthesis can be catalyzed by several homologous genes. The enzyme *fabH2* β-ketoacylACP synthase is a key to the initiation of branched-chain fatty acid biosynthesis in particular (Meng et al., 2018). It is worth noting that the homologous gene, *fabH1*, responsible for the initiation of straight chain fatty acid biosynthesis was detected in our cultures, but it was not differentially expressed. This result indicates that branched chain fatty acid synthesis, initiated by *fabH2* (LFC 0.75), may have been favored over straight chain fatty acids. It is interesting to note that an increase in the amount of branched fatty acids relative to straight chain and unsaturated fatty acid substituents in intact polar lipids was demonstrated in other unpublished work in preparation conducted on *S. oneidensis* at ~150 MPa for 15 min indicating that a LFC threshold of >1 may not capture all the essential changes in function relevant to the experiment.

### 4.2 Amino acid biosynthesis

In this study, genes associated with amino acid metabolism, specifically arginine biosynthesis, were highly upregulated (Figures 3, 4). Arginine is synthesized in eight enzymatic steps in *E. coli* (Charlier and Bervoets, 2019), four of which were upregulated in this study (*argA*, LFC 2.7*; argB*, LFC 4.6; *argC* LFC 4.5; *argF*, LFC 4.0). The need for additional arginine specifically is unclear, as the TSB growth medium is abundant in all the necessary amino acids and no other amino acid biosynthesis pathways were upregulated. Arginine production has not been observed previously as a response to HHP, however, arginine and its intermediate ornithine play a role in protecting cells against acidity or oxidative stress (Bearson et al., 2009; Charlier and Bervoets, 2019), both of which could be enhanced by HHP. In addition, the arginine transcriptional regulator gene *argR* was upregulated (LFC 1.9). This regulator responds to the presence of arginine and represses the expression of the arginine biosynthesis enzymes (Cho et al., 2012). We speculate that this provides evidence for arginine overproduction at the metabolite level, and that ArgR was upregulated post-treatment. It may be the case that the arginine biosynthesis genes would be reduced in expression after additional recovery time at ambient post-treatment, especially if the main function of the arginine was a response to HHP stress. More work is needed to determine the specific role of these upregulated arginine biosynthesis genes in response to HHP. The singling out of the arginine biosynthesis pathway as well as the degree to which it was upregulated leads to the conclusion that arginine may play an important role in resistance to pressures >150 MPa.

Positively charged (basic) amino acids such as arginine have been found to be overrepresented in piezophilic proteomes. Peoples et al. (2020) found that piezophilic Gammaproteobacteria have a more basic proteome than non-piezophilic Gammaproteobacteria. Di Giulio (2005) computationally compared a random sample of 141 orthologous proteins from the piezophile *Pyrococcus abyssi* and the non-piezophile *Pyroccus furiosus* and found that arginine is the most piezophilic amino acid, with the accumulation of arginine favored at the expense of other amino acids in the piezophilic organism. Nath and Subbiah (2016) used machine learning algorithms to discriminate between piezophilic and non-piezophilic protein sequences and determined that polar amino acids were more prevalent in piezophiles. The increase in polar amino acids is thought to increase the potential for weak chemical interactions and stabilize bonds at high-pressure (Mrabet et al., 1992; Nath and Subbiah, 2016). While it is unlikely that arginine would have been incorporated in the proteome at this experimental timeline, the similarity of this response to that of piezophiles leads to a hypothesis that arginine could be serving a similar stabilizing purpose for *S. oneidensis* at HHP. Experimental verification is needed to confirm whether arginine upregulation is observed at the metabolite level in addition to the gene expression level, and to clarify the function of arginine overproduction at HHP in *S. oneidensis*, as this is the first time arginine upregulation has been observed in a non-piezophile in response to HHP.

### 4.3 Common adaptation mechanisms to environmental stressors

It is thought that microorganisms have adapted a common strategy to cope with various stressors such as pressure, temperature, salinity, oxidation, osmotic stress, or pH extremes (Ambily Nath and Loka Bharathi, 2011; Zhang et al., 2015). This common adaptation strategy may be in part due to the similarity in damage each of these environmental stressors causes on the cell (Iwahashi et al., 1993). Both piezophiles and non-piezophiles may employ these common strategies during the “fine-tuning” of their gene expression in response to pressure. *S. oneidensis* MR-1 was first isolated from Lake Oneida in New York state, a lake with an average depth of about 6.7 m, making it unlikely the pressure response of MR-1 is due to prior pressure exposure. However, Lake Oneida does experience frequent temperature variations and freeze/thaw cycles, and MR-1 has been shown previously to grow at temperatures as low as 3°C (Abboud et al., 2005). The observed pressure resistance of *S. oneidensis* under HHP stress may therefore have been enhanced by existing adaptations needed to withstand other environmental stressors present within the isolation environment, such as antioxidant defense or cold shock proteins.

Synergistic effects between pressure and other stressors have been noted, as organisms pre-treated or simultaneously treated with various stressors such as high or low temperature or increased salinity tend to withstand higher pressures upon further exposure (Casadei et al., 2002; Scheyhing et al., 2004; Takai et al., 2008; Scoma et al., 2019; Wang et al., 2021). The accumulation of low molecular weight osmolytes, such as glutamate and trimethylamine N-oxide (TMAO), is one mechanism that cells use to cope with a variety of extremes such as cold, heat, salinity, pH, and high pressure (Yancey, 2005; Gaussier et al., 2021). Though we did not test for the accumulation of these solutes here, *S. oneidensis* upregulated several genes common to environmental extremes such as heat, cold, and oxidative stress.

Both heat-shock proteins (HSP) and cold-shock proteins (CSP) are known to be induced in response to HHP (Ambily Nath and Loka Bharathi, 2011). The cold-shock proteins CspG and CspC are homologs of the first cold-shock protein reported, CspA (Goldstein et al., 1990; Phadtare and Inouye, 2008). In this study, *cspG* (1.7 LFC) was upregulated while *cspC* (-1.3 LFC) was downregulated (Supplementary Table S2). Low temperatures stabilize the secondary structures of RNA, which is presumed to slow down transcription and translation (Phadtare, 2004). The homologs CspA, CspB, CspG, and Cspl are induced by cold temperatures (15°C) and act as RNA chaperones to melt the secondary structures in nucleic acids and facilitate cold-shock transcription and translation (Phadtare and Inouye, 2008). While the homologs CspE and CspC may aid in the cold-shock response in *E. coli*, they are not induced by cold temperatures (Phadtare, 2004). We speculate that *cspG* may have been recruited here to aid in the transcription and translation processes that was impaired due to HHP.

The expression of the major HSP DnaK (molecular chaperone HSP70) increased by a LFC of 0.8. Gene *DnaK* is a commonly upregulated gene in response to HHP conditions. Aertsen et al. (2005) found that *dnaK* expression increased in response to pressure and plays a protective role. The DnaK protein has been found for *L. sanfransicanis* under HHP, low pressure, and high NaCl stress conditions (Hörmann et al., 2006). Sato et al. (2015) found that the expression of HSPs is increased in piezophile *S. violacea*, even at optimal growth conditions (30 MPa), and that they play essential roles in maintaining protein complex machinery, including ribosomes, as well as growth and viability at HHP. The repeated observation of CSPs and HSPs in both piezophiles and non-piezophiles at HHP provides further evidence that they are not specific to one environmental extreme contrary to what their names might suggest.

Genes involved in antioxidant defense were highly upregulated in our HHP treated *S. oneidensis* including catalase *cat_1* (LFC 3.3) and the hydrogen peroxide–inducible genes activator *oxyR* (LFC 2.3). Antioxidant defense mechanisms have been shown to be important at HHP. Wang et al. (2021) performed transcriptomic analysis on *Sporosarcina psychrophila* DSM 6497 to compare gene expression at 50 MPa with and without the addition of 0.25 M MgCl_2_. Antioxidant defense systems, including the enzyme catalase, were found to be upregulated in response to the addition MgCl_2_ and was thought to enhance the growth capability of the *S. psychrophila* at 50 MPa (Wang et al., 2021). A mutant of *Shewanella piezotolerans* WP3 with enhanced antioxidant defense mechanisms also showed improved growth at 20 MPa compared to the wild type *S. piezotolerans* (Xie et al., 2018). These results indicate that *S. oneidensis* adapted to short-term HHP in part by drawing upon stress response genes typically activated under conditions of heat, cold, and oxidative stress.

### 4.4 Survival and potential for evolution

It is important to first distinguish high pressure survival from high pressure growth. As of this writing, no study has demonstrated growth *in situ* at or above 150 MPa. While we cannot yet prove that *S. oneidensis* was capable of replicating under pressure, our transcriptomic results indicate that the organism was metabolically active *in situ*.

Experiments with surviving cells had intact cell numbers post treatment similar to their untreated control counterparts (Figure 2). Additionally, visual observation of the unopened experimental bags post treatment confirmed that counted intact cells were not dormant/inactive or a result of contamination introduced after the bags were opened, as unopened bags increased in turbidity post-treatment. Similar survival rates after exposure to 150 MPa for 5 min have been observed for *E. coli* O157:H7 (Zhu et al., 2021). Results of this study indicate that *S. oneidensis* is more pressure resistant than the *E. coli* strain tested in previous work, as RNA-sequencing analysis after 150 MPa for 5 min found that fifteen genes were downregulated and only one gene was upregulated indicating impaired cellular activities in response to HHP (Zhu et al., 2021). Here, we have shown that the majority of DEGs are upregulated, and that absolute values of upregulation are higher than downregulation (Figures 3, 4), indicating that *S. oneidensis* engages in an active response to HHP.

It has been observed that “training” steps, in the form of exposing a culture to high pressures up to 1.5 GPa for a short period of time and allowing it to recover and divide at ambient pressures before returning the culture to a higher pressure, improves yield of colony forming units (CFUs) post-depressurization (Vanlint et al., 2011; Hazael et al., 2014); however, these studies did not show that organisms were capable of replicating *in situ* at these pressures. In our study, *S. oneidensis* MR-1 with no prior pressure exposure could modify its biological functions at the transcriptome level in response to 15 min at 158 MPa. This finding provides evidence that pressures at the top of Titan's subsurface ocean would not necessarily inhibit the evolution of life, as *S. oneidensis* survived HHP exposure and therefore survivors may evolve under such conditions with repeated/more prolonged exposure. DNA repair genes such as *lexA* (LFC 2.9)*, dinB* (LFC 2.1)*, dinG* (LFC 2)*, recA* (LFC 1.3)*, recN* (LFC 4) and*, recO* (LFC 1.3) were upregulated, indicating HHP may cause DNA damage, which we speculate could increase the mutation rate (Galhardo et al., 2007). The genes *lexA* and *recA*, which are involved in the so-called “SOS response” characterized by a rapid increase in DNA repair capacity (Welch et al., 1993), have also been found to be upregulated in *E. coli* in response to HHP (Aertsen et al., 2004). Though the genome of the HHP *S. oneidensis* survivors was not sequenced, some mutation may have been preserved in the surviving post-treatment population. Survivors of HHP treatment will be used in future work to attempt to evolve a piezophilic strain capable of replicating at Titan-relevant pressures.

The evolution of more pressure resistant biomolecules is a key differentiator of piezophiles as certain biomolecules of non-piezophiles may become non-functional at some pressure thresholds. It has been previously shown that RNA polymerase, an enzyme responsible for transcribing DNA to RNA, was dissociated at 140 MPa in *E. coli* but not in the piezophile *Shewanella violacea* (Kawano et al., 2004), indicating that organisms that evolve in the presence of HHP possess specialized transcriptional machinery. The decreased functionality of key biomolecules (i.e., RNA polymerase) in *S. oneidensis* in this study could explain the observed decrease in transcription after 2 h of HHP incubation (Supplementary Figure S4B), despite the observed intact cell membranes (Supplementary Figure S2). Future work should investigate the potential convergence in proteins from lab-evolved piezophiles and piezophiles in nature.

It may be possible to adapt a non-piezophile to Titan-like pressure conditions via adaptive laboratory evolution (ALE); a strategy that mimics natural selection to obtain new microbial strains using artificial selective pressure to expedite the accumulation of beneficial mutations (Wu et al., 2022). Marietou et al. (2015) used ALE to adapt a piezophilic strain of *E. coli* that could grow more efficiently *in situ* at 60 MPa than at atmospheric pressure. Additional challenges come with ALE at Titan-like pressures, however, as pressures > 100 MPa are known to cause significant damage to cellular components (Mackey and Mañas, 2008; Gayán et al., 2017). Hauben et al. (1997) observed that *E. coli*, after 18 cycles of pressurization to 280 MPa and recovery at atmospheric pressure, showed an enhanced survival rate compared to untrained strains when exposed to 800 MPa. However, the *E. coli* strain that acquired this enhanced survival at 800 MPa did not acquire any enhanced growth ability at pressures even as low as 50 MPa (Hauben et al., 1997). This finding raises the possibility that adaptation to short-term HHP exposure would not resemble that of growth under the same HHP. Outside the realm of HHP, another study by Lin et al. (2013) compared the relationship between the transcriptomic response of a wild-type *Thermoanaerobacter* to that of a laboratory evolved, 6% ethanol tolerant strain. This study suggested convergence (i.e., similarity) in the transcriptomic response between the evolved strain and the wild-type strain to acute (4 h) exposure to stressful ethanol concentrations of 0.15%. More research is needed to determine the feasibility of ALE for Titan-relevant pressures and the relationship between the short-term HHP response to that of a laboratory evolved strain.

### 4.5 Astrobiological significance

The diversity of environments and associated life within Earth's ocean allows for extrapolations to other ocean-bearing worlds (Hendrix et al., 2019; Merino et al., 2019; Miller et al., 2023). The molecular adaptations that define the genomes, lipidomes, and proteomes of extremophiles may be useful as biosignatures (Georgiou, 2018; Mudge et al., 2021). Particularly relevant to ocean worlds will be membrane adaptations and amino acid differences as these will likely be more stable and detectable by instruments aboard space missions (Neveu et al., 2017). Here, we have shown that a non-piezophilic bacteria can tolerate the high pressures expected in the upper part of Titan's ocean. Our study suggests that Titan life could require (1) adapted membrane structures to allow for the necessary membrane fluidity, (2) enhanced DNA repair mechanisms to compensate for potential HHP-induced degradation, and (3) increased proportion of polar amino acids. Additional studies conducted at Titan-like pressures of > 150 MPa on evolutionarily distinct organisms are needed to enhance our understanding of the adaptations necessary to withstand such pressures. Adaptations that are found to be universal among piezophiles that have evolved under HHP for millions of years and to non-piezophiles exposed to acute, short-term HHP shocks, would inform the adaptations that potential life on Titan would need to cope with HHP.

Beyond pressure, potential life on Titan would also need to overcome additional stress sources such as the possible presence of ammonia and cold temperatures (Sotin et al., 2021). Organisms adapted to multiple extremes such as cold temperatures and high pressures have been found, such as the microbial ecosystems deep in glaciers on Earth (up to ~26 MPa; e.g., Miteva and Brenchley, 2005; Christner et al., 2014). Characterization of the survival strategies developed by organisms in response to combinations of stressors relevant to Titan would help further clarify the potential for life on ocean worlds.

### 4.6 Challenges and opportunities in HHP research

The difficulties associated with laboratory experimentation has made HHP an underexplored constraint on microbial physiology and evolution (Xiao et al., 2021). Research at pressures relevant to Titan presents unique challenges, particularly in the realm of reproducibility. High pressure equipment varies from one laboratory to another (Macdonald, 2021b), and to our knowledge, there is no instrumentation capable of *in situ* sampling above ~100 MPa with the experimental volumes needed for DNA, RNA, and lipid extractions. This means that, with current systems, a microbial culture must undergo decompression before sampling, which potentially influences the subsequent analyses and results. In addition, faster rates of decompression increase cell damage (Park and Clark, 2002; Cario et al., 2022).

The decompression rates used here (45.1 ± 17.7 MPa/min) were tested prior to the reported experiments and were determined not to cause significant cellular rupture, and indeed, the numbers of recovered intact cells remained similar to those from the untreated control (Figure 2). However, we cannot exclude the possibility that a subset of the gene expression changes observed in this study was a response to the decompression process. Nonetheless, several of the response mechanisms observed here are similar to those previously identified in piezophiles and/or non-piezophiles exposed to lower pressures, which supports that the adaptations observed here are indeed a likely result of the HHP variable. Unfortunately, pressurization and decompression rates are not consistently reported in the literature, and such data would be imperative to improve experimental reproducibility.

As mentioned before (Section 4.3), synergistic effects between pressure and other “stressors” such as high temperatures, low temperatures, and salinity variations have been observed (Casadei et al., 2002; Scheyhing et al., 2004; Takai et al., 2008; Scoma et al., 2019; Wang et al., 2021). For example, the addition of MgCl_2_ to a bacterial culture improved its pressure tolerance at 50 MPa (Wang et al., 2021). Previous work has also found that genetic similarity is a poor indicator of HHP tolerance, as genetically similar strains can have vastly different responses to HHP treatment (Malone et al., 2006; Miller et al., 2023), and factors such as bacterial growth phase can influence results from a given strain (Griffin et al., 2011). Even within our own study, significant differences in survival were observed between *S. oneidensis* cultures that came from the same parent population (Figure 2). These synergistic effects and physiological differences should be the subject of future work, as a particular combination of experimental parameters could have the potential to extend the currently understood HHP limit of life.

In this work, the success of the 2 h experiments may have been limited by the incubation of cells in stationary phase. This method was used because it was determined that stationary phase cells are more pressure resistant than those in exponential phase (e.g., Mackey and Mañas, 2008; Griffin et al., 2011). While not as relevant during incubations on a short time scale, longer-term pressure incubations may risk being confounded as the cells enter a less active state. Therefore, pressure incubations with the goal of long-term HHP survival or growth may be more successful with cells in exponential phase. Additional “training” steps (section 4.4) may be necessary as non-piezophiles are more sensitive to pressure in exponential phase.

## 5 Conclusions

*S. oneidensis* MR-1 was subjected to short-term hydrostatic pressure of 158 MPa to understand its mechanisms of survival at the pressure expected near the top of Titan's subsurface ocean. We have found that *S. oneidensis* MR-1 with no prior pressure exposure was capable fine tuning its gene expression in response to 15 min at 158 MPa. Our results indicate that the organism was metabolically active *in situ* for this short experimental period. A long term, 2 h experiment at 158 MPa was also attempted, and we show *S. oneidensis* MR-1 can survive Titan relevant pressures over longer time periods with minimal pressure training, although experimental design challenges remain. Future work is needed to determine if evolution at HHP relevant to Titan is possible in a laboratory setting. Additionally, only 46% of the differentially expressed genes (DEGs) could be annotated and the biological purpose for many of the annotated DEGs has not yet been determined. These transcripts should be the subject of further investigation to fully uncover the short term response of *S. oneidensis* to HHP. Potential life on Titan, as with life on Earth, would exist within the bounds of physical and chemical principles. As far as pressure is concerned, part of Titan's ocean could be habitable to microorganisms using adaptations already demonstrated by terrestrial organisms. Adaptations to pressure included increases in genes encoding the biosynthesis of the polar amino acid arginine, the regulation of cold- shock proteins, and the regulation of fatty acid chain lengths, among others. As these mechanisms are common to piezophiles and non-piezophiles alike, there is reason to believe potential life in Titan's high pressure environments may utilize similar adaptations.

## Data availability statement

The data presented in the study are deposited in the SRA repository, accession number PRJNA1010834. The data has been released and can be viewed at the following link: https://www.ncbi.nlm.nih.gov/bioproject/PRJNA1010834.

## Author contributions

JM: Conceptualization, Formal analysis, Investigation, Methodology, Visualization, Writing—original draft, Writing—review & editing. DR: Investigation, Writing—review & editing. OB: Methodology, Writing—review & editing. MM: Conceptualization, Funding acquisition, Writing—review & editing. RL: Funding acquisition, Project administration, Writing—review & editing. FK: Conceptualization, Funding acquisition, Methodology, Supervision, Writing—review & editing. D'AM-D: Conceptualization, Funding acquisition, Methodology, Supervision, Writing—review & editing.
