# Supplementary material for: Biological functions at high pressure: transcriptome response of Shewanella oneidensis MR-1 to hydrostatic pressure relevant to Titan and other icy ocean worlds
Source: Front Microbiol. 2024 Feb 13;15:1293928. doi: 10.3389/fmicb.2024.1293928 (PMC10896736; doi:10.3389/fmicb.2024.1293928)
Supplement: Supplementary file 4 [file Image_1.pdf]

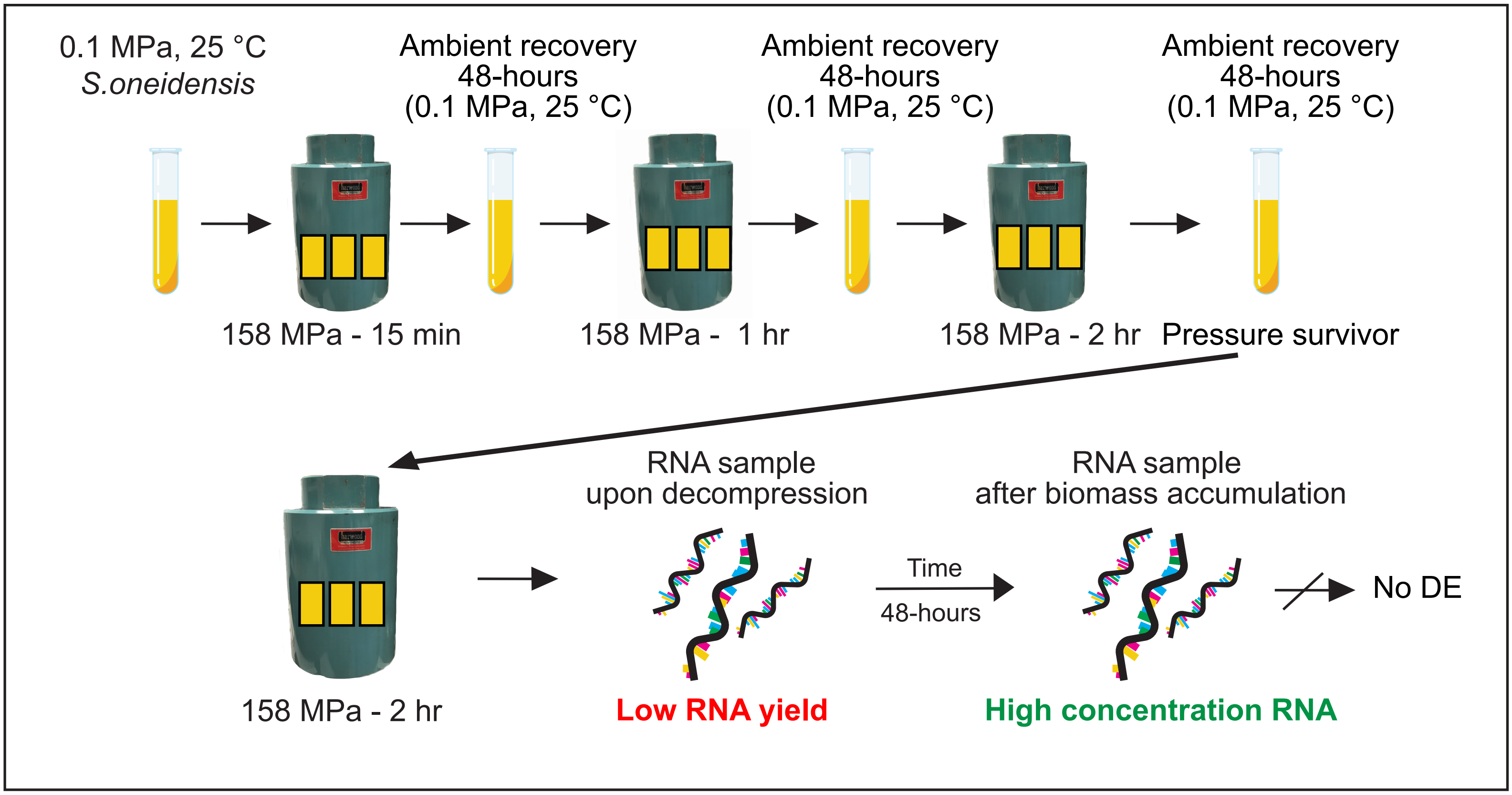

Figure S1. Experimental setup for the 158 MPa – 2 hour treatment. *S. oneidensis* was subjected to successive pressure cycles, each at 158 MPa but of increasing duration (15 minutes, 1 hour, 2 hours), and each followed by recovery at ambient pressure for 48 hours without re-inoculation or dilution with additional TSB media. After recovery, the pressure treated cell cultures were re-inoculated into fresh TSB media (1:1000 dilution) and grown to early stationary phase before the next pressure cycle.
