# Supplementary material for: Biological functions at high pressure: transcriptome response of Shewanella oneidensis MR-1 to hydrostatic pressure relevant to Titan and other icy ocean worlds
Source: Front Microbiol. 2024 Feb 13;15:1293928. doi: 10.3389/fmicb.2024.1293928 (PMC10896736; doi:10.3389/fmicb.2024.1293928)
Supplement: Supplementary file 5 [file Image_2.pdf]

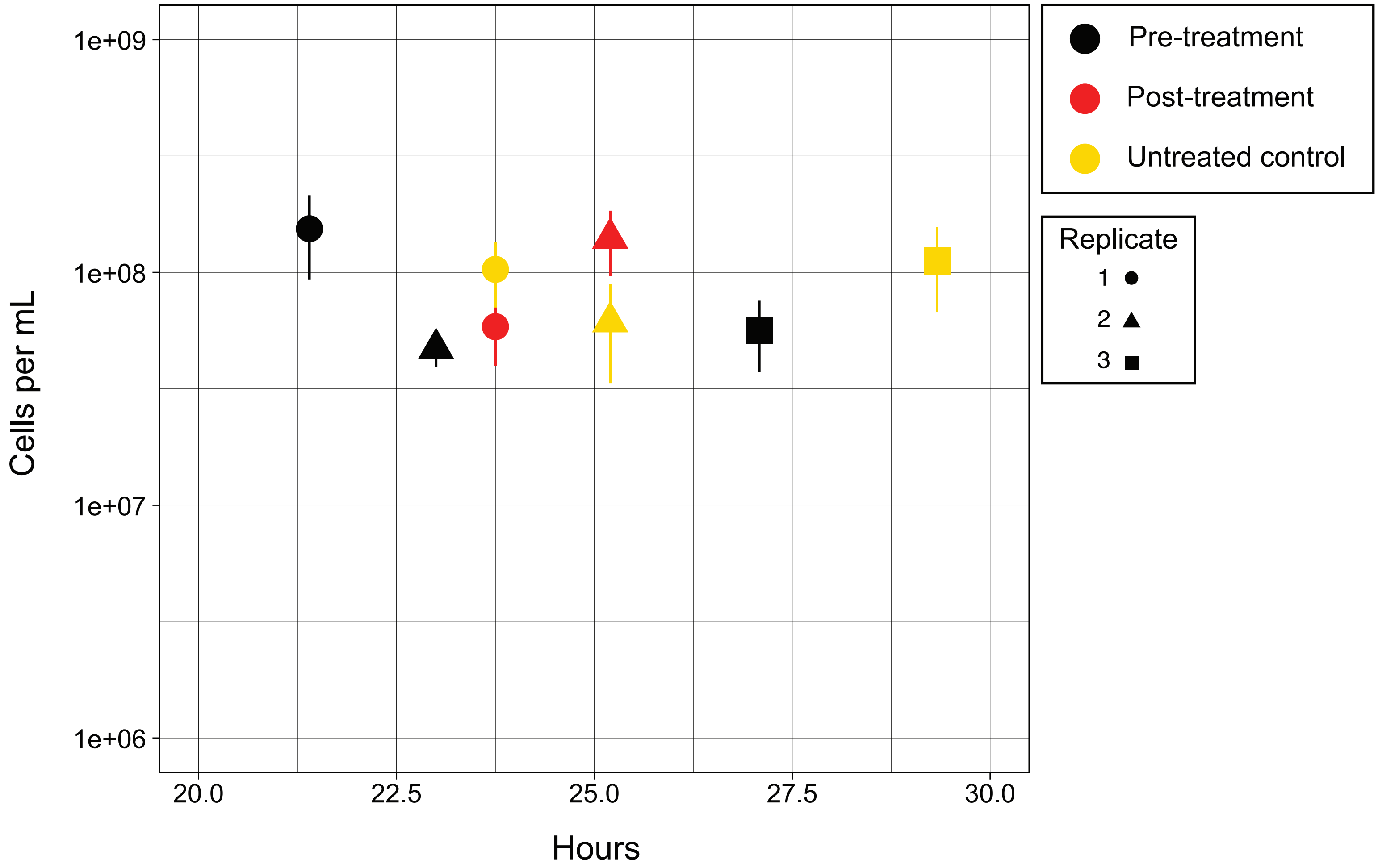

Figure S2. Cell counts of DAPI stained cells for the 150 MPa – 2 hour treatments. Error bars represent standard deviation about the mean least 10 fields of view were counted from each filter. Shapes indicate experimental replicates. Colors indicate treatment phase; initial cultures (black) were transferred into FEP bags and either maintained at ambient conditions (yellow) or subjected to 158 MPa treatment for 2 hours minutes (red shapes). No intact cells were recovered from the third replicate (squares) of this treatment.
