# Supplementary material for: Biological functions at high pressure: transcriptome response of Shewanella oneidensis MR-1 to hydrostatic pressure relevant to Titan and other icy ocean worlds
Source: Front Microbiol. 2024 Feb 13;15:1293928. doi: 10.3389/fmicb.2024.1293928 (PMC10896736; doi:10.3389/fmicb.2024.1293928)
Supplement: Supplementary file 6 [file Image_3.pdf]

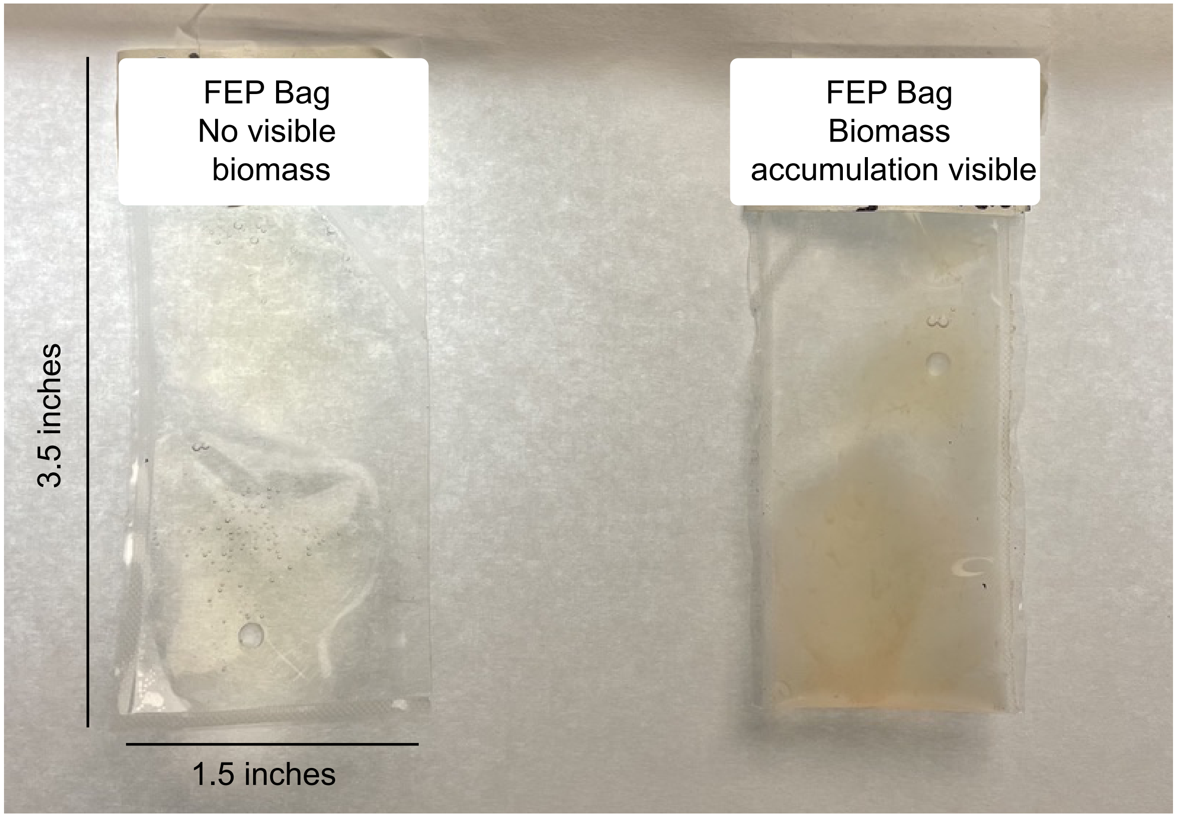

Figure S3: Image shows FEP bags inoculated with *S. oneidensis.* Left bag shows an example of a post-treatment bag deemed nonviable (light color from Tryptic Soy Broth), while the right bag shows a post-treatment bag with biomass accumulation (increased turbidity and change in color). Images taken 48-hours post decompression from different 158 MPa - 2 hours experiments.
