# Supplementary material for: Biological functions at high pressure: transcriptome response of Shewanella oneidensis MR-1 to hydrostatic pressure relevant to Titan and other icy ocean worlds
Source: Front Microbiol. 2024 Feb 13;15:1293928. doi: 10.3389/fmicb.2024.1293928 (PMC10896736; doi:10.3389/fmicb.2024.1293928)
Supplement: Supplementary file 7 [file Image_4.pdf]

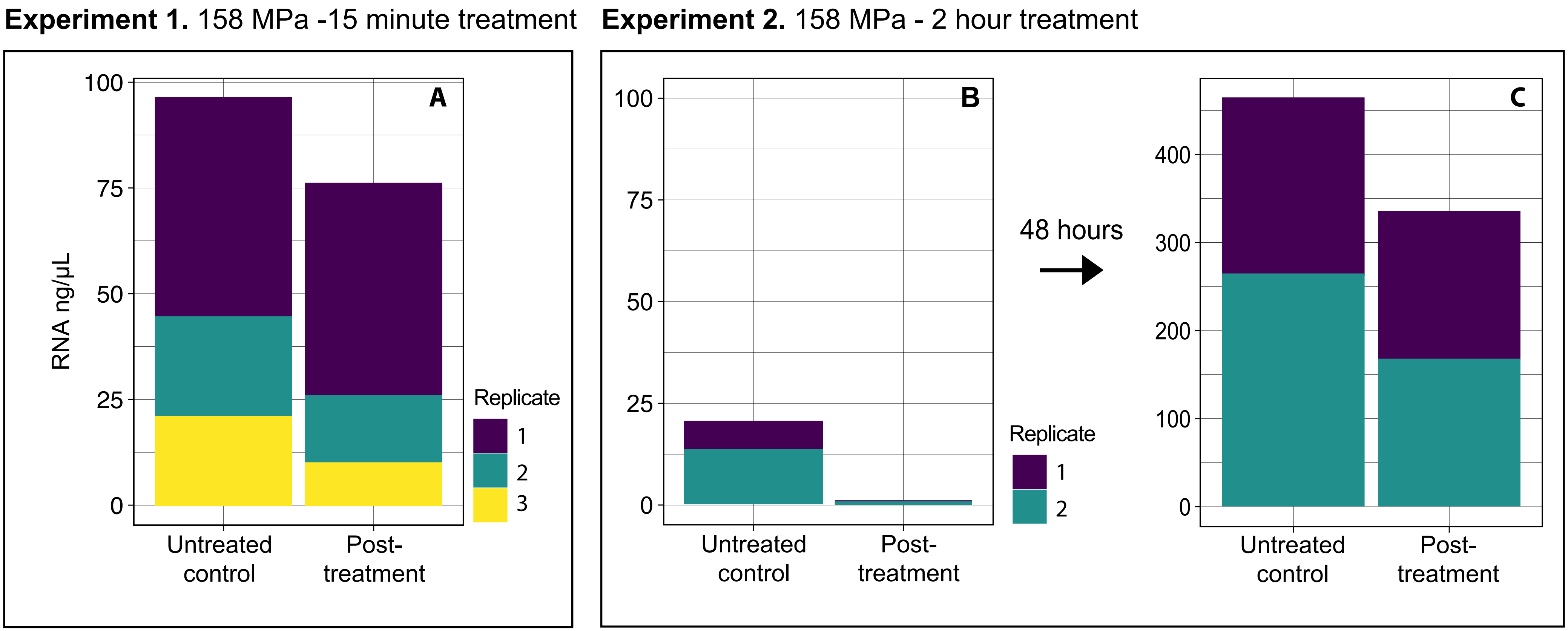

Figure S4. A) RNA concentration post-high pressure treatment of 158 MPA for 15 minutes compared to untreated controls. Samples were taken upon decompression. B) RNA concentration post-high pressure treatment of 158 MPA for 2 hours compared to unpressurized controls. The third replicate is not shown because no intact cells were detected post decompression. C) RNA concentration after 48 hours of incubation at ambient conditions post-decompression from the 2-hour experiments.
