# Supplementary material for: Biological functions at high pressure: transcriptome response of Shewanella oneidensis MR-1 to hydrostatic pressure relevant to Titan and other icy ocean worlds
Source: Front Microbiol. 2024 Feb 13;15:1293928. doi: 10.3389/fmicb.2024.1293928 (PMC10896736; doi:10.3389/fmicb.2024.1293928)
Supplement: Supplementary file 8 [file Image_5.pdf]

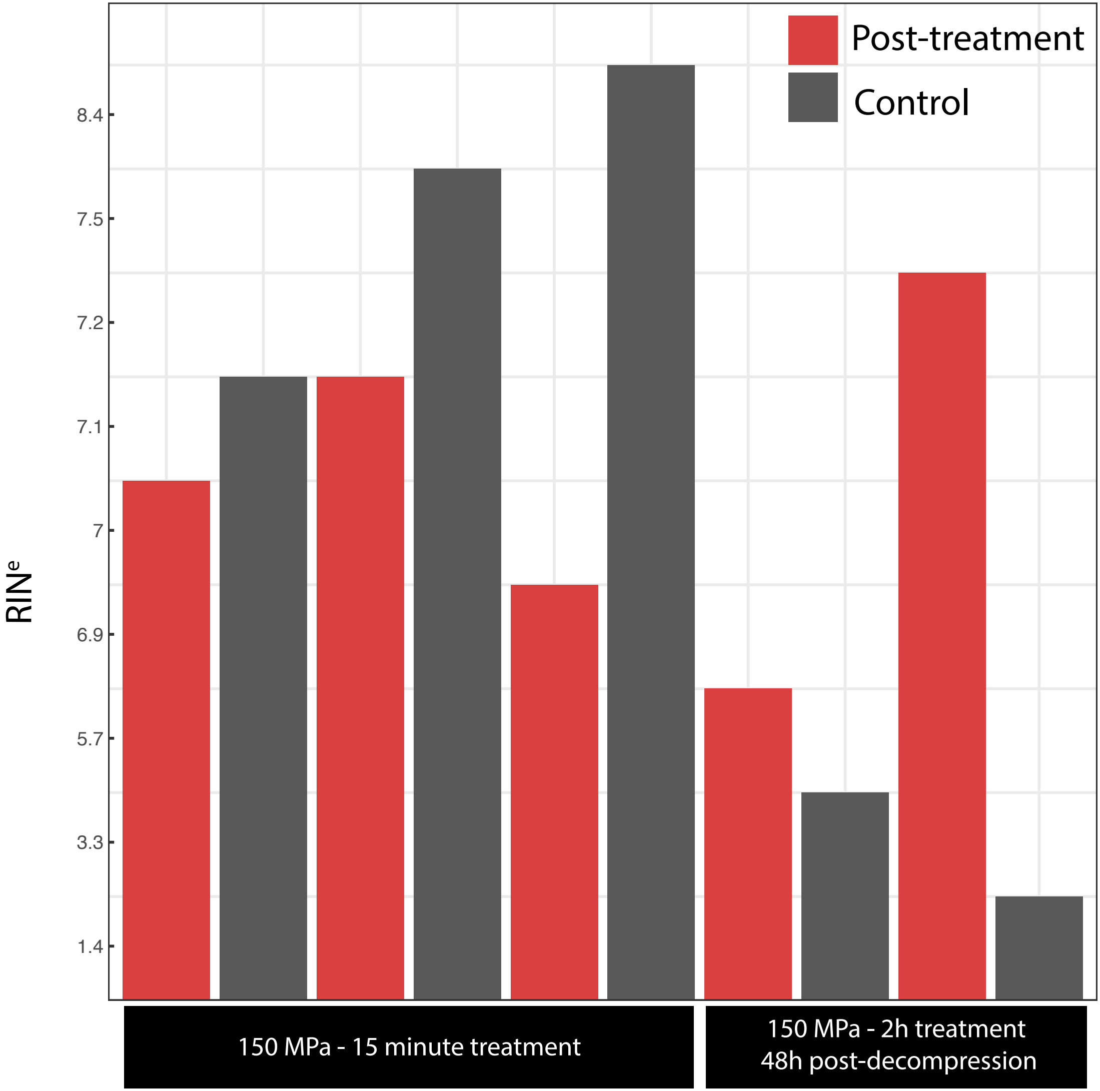

Figure S5. RNA integrity numbers (RIN^e) for all samples. Samples from the 15 minute experiment have RIN^e values >6.9 and were appropriate for use in differential expression. RIN^e values for the control group 2 hour treatment (gray bars)were <5.5 which indicated the RNA was too degraded for use in differential expression analyses.
