# Supplementary material for: Biological functions at high pressure: transcriptome response of Shewanella oneidensis MR-1 to hydrostatic pressure relevant to Titan and other icy ocean worlds
Source: Front Microbiol. 2024 Feb 13;15:1293928. doi: 10.3389/fmicb.2024.1293928 (PMC10896736; doi:10.3389/fmicb.2024.1293928)
Supplement: Supplementary file 9 [file Image_6.pdf]

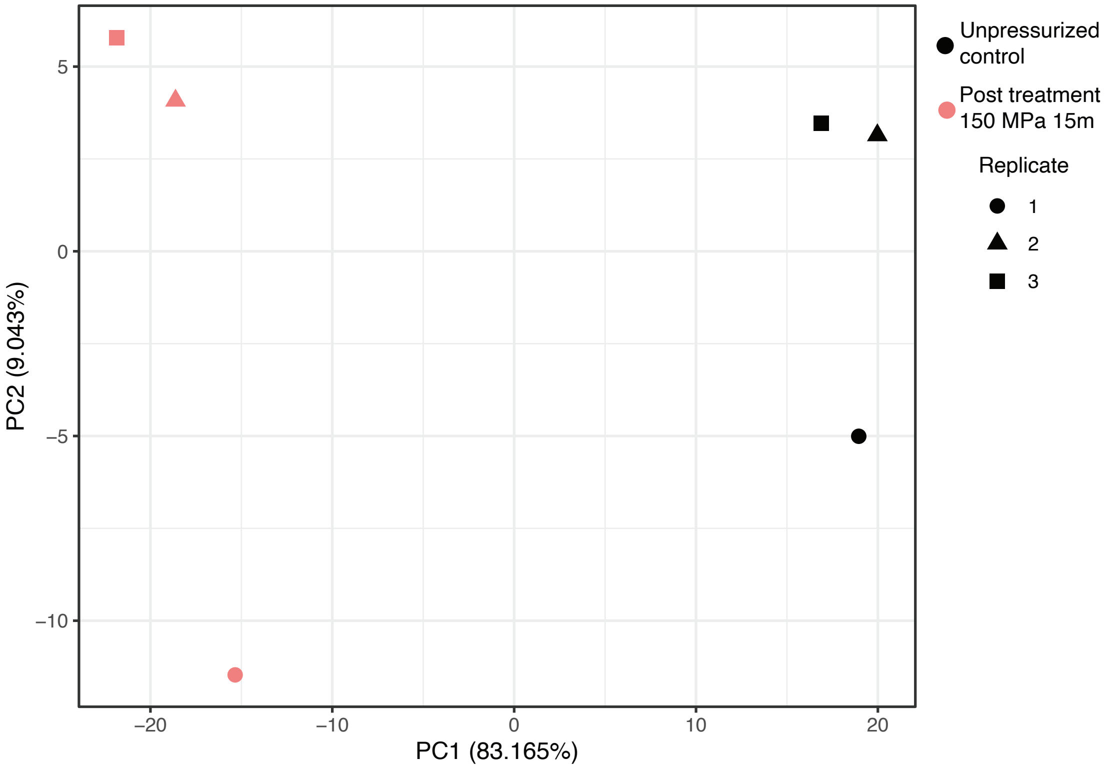

Figure S6. Principal components analysis (PCA) was conducted with the CPM normalized expression of the 1204 genes used for differential expression analysis. Black shapes represent untreated controls and pink shapes represent pressure treated samples. The first two principal components explained >90% of the variance between samples, with much of the variance being explained by PC1 (83%).
